# Isolated Skin Metastasis From Papillary Thyroid Carcinoma: A Rare Presentation

**DOI:** 10.1210/jcemcr/luaf106

**Published:** 2025-05-15

**Authors:** Naseem Eisa

**Affiliations:** Community Health Partners, Fresno, CA 93720, USA

**Keywords:** papillary thyroid carcinoma, skin metastasis, thyroid cancer, rare case, metastatic thyroid carcinoma

## Abstract

Papillary thyroid carcinoma (PTC) is the most common thyroid malignancy and is often characterized by a high rate of regional lymph node involvement. However, distant metastasis is uncommon, and cutaneous metastases are particularly rare, occurring in fewer than 0.1% of cases. This report presents an unusual case of isolated skin metastasis as the first manifestation of distant spread in PTC. The case highlights the diagnostic challenges associated with this rare presentation, explores potential mechanisms underlying cutaneous dissemination, and underscores the significance of a multidisciplinary approach to management. The findings emphasize the importance of long-term surveillance and meticulous pathological evaluation in patients with PTC, particularly in cases with atypical metastatic patterns.

## Introduction

Papillary thyroid carcinoma (PTC) is the most prevalent form of thyroid malignancy, accounting for approximately 80% to 90% of all thyroid cancer cases [1]. Although the prognosis for PTC remains favorable, with a 10-year survival rate exceeding 90%, a subset of patients experiences distant metastases, which occur in approximately 4% to 23% of cases [2]. The lungs, bones, and central nervous system represent the most common sites of metastatic involvement. In contrast, cutaneous metastases are exceedingly rare, occurring in fewer than 1% of cases [2], and are often associated with advanced or widely disseminated disease. Skin metastases from PTC typically involve regions with a rich vascular supply, such as the head, neck, and scalp. These lesions may present as erythematous, violaceous, or flesh-colored nodules, which can be asymptomatic or associated with pruritus, ulceration, or pain [3, 4]. The latency period between the primary diagnosis of PTC and the subsequent development of skin metastases can range from several months to decades, with reported median intervals of 8 to 30 years [5]. Given the rarity of this phenomenon, histopathological confirmation with immunohistochemical staining for thyroid transcription factor-1 (TTF-1) and thyroglobulin (Tg) is critical for an accurate diagnosis [2]. This report presents a rare case of isolated cutaneous metastasis occurring 5 years after the initial treatment of PTC. The case underscores the importance of long-term surveillance in patients with thyroid malignancies and highlights the diagnostic and therapeutic complexities associated with this unusual presentation.

## Case Presentation

A 75-year-old female with a history of arthritis and osteopenia was referred for evaluation of a thyroid nodule. The patient denied any history of smoking, alcohol or drug abuse, exposure to irradiation, or family history of thyroid cancer. Additionally, she reported no symptoms of compression. On physical examination, a palpable nodule was detected in the right thyroid lobe.

## Diagnostic Assessment

**Figure 1.**
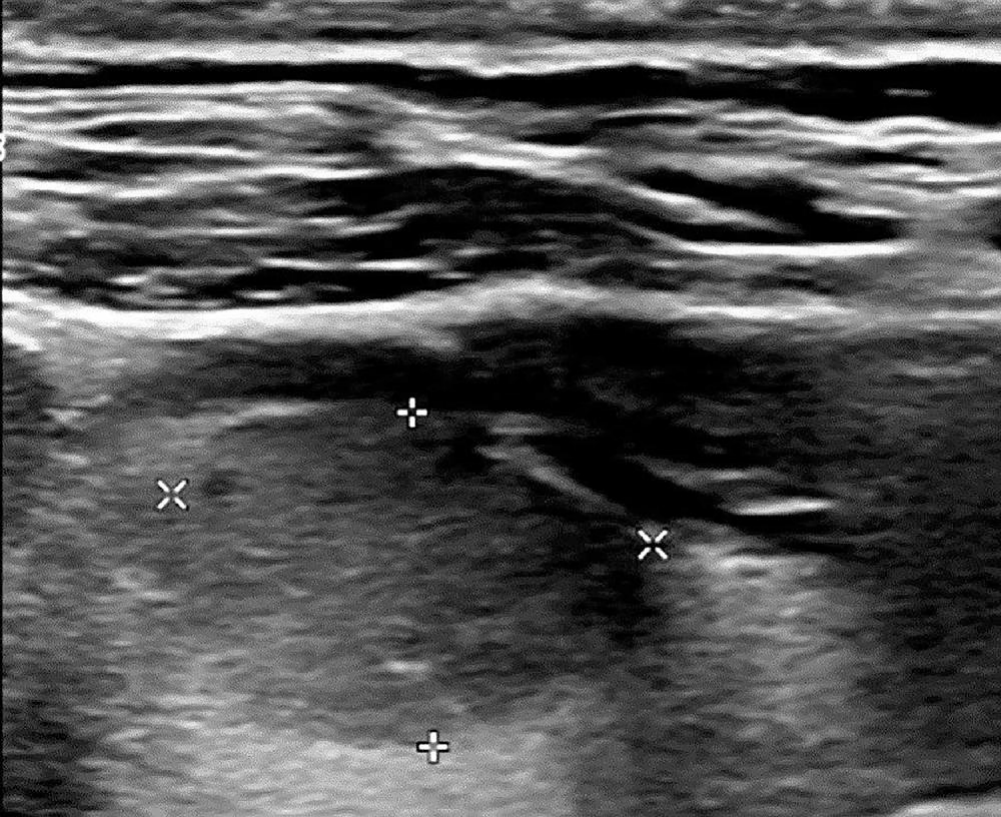
Neck ultrasound reveals a 1.1 × 1.5 × 1.9 cm nodule in the right lobe with the following characteristics based on the ACR TI-RADS: solid composition (2 points), hypoechoic echogenicity (2 points), wider-than-tall shape (0 points), smooth margin (0 points), and no echogenic foci (0 points). The total score is 4 points, classifying the nodule as TI-RADS 4. Abbreviations: ACR-TI-RADS, American College of Radiology Thyroid Imaging Reporting and Data System.

Neck ultrasound at initial presentation identified a hypoechoic nodule in the right thyroid lobe, measuring 1.1 × 1.5 × 1.9 cm. The lesion exhibited a solid composition, a wider-than-tall shape, and smooth margins without echogenic foci. According to the American College of Radiology's Thyroid Imaging Reporting and Data System, the nodule is classified as Thyroid Imaging Reporting and Data System 4 (Fig. 1). No lymphadenopathy was detected in the central or lateral compartments of the neck. Fine-needle aspiration biopsy confirmed the diagnosis of PTC, classical variant, classified as Bethesda category 6.

The patient subsequently underwent a total thyroidectomy with prophylactic central neck dissection, including levels VI and VII, without complications. Histopathological evaluation revealed a 2 × 2 × 1.5 cm encapsulated classical variant of PTC and a 2 mm unencapsulated follicular variant of PTC, both confined to the right lobe. The classical variant exhibited capsular invasion with focal involvement of the specimen margin. Bilateral lymphocytic thyroiditis was noted. No lymphovascular invasion or extrathyroidal extension was observed. Eight lymph nodes were resected, all of which were benign. No parathyroid tissue was identified.

Based on the American Thyroid Association risk stratification system, the tumor was classified as low-risk for recurrence, and a shared decision-making approach was taken not to proceed with radioactive iodine (RAI) ablation. The initial TSH goal was to be maintained in the mid to lower half of the reference range (normal range: 0.5-3.0 mU/L or 0.5-3.0 µIU/mL). Instead, the patient was placed under periodic surveillance, during which serial neck ultrasounds and Tg assessments remained negative, showing no evidence of recurrence and indicating an excellent response to therapy (Table 1).

**Table 1.** Postthyroidectomy follow-up surveillance data

Time after TT	TSH value*^a^*	Tg value*^b^*	TgAb value*^c^*	Neck US	PET/CT imaging	Excision of the skin lesion and two adjacent lymph nodes	RAI-131 ablation	WBS post-RAI-131 ablation	Non-contrast Chest CT
**6 weeks**	0.19 (mU/L or µIU/mL)	<0.1 (µg/L or ng/mL)	≤1 (IU/L or IU/mL)	Negative					
**3 months**	0.29 (mU/L or µIU/mL)	<0.1 (µg/L or ng/mL)	≤1 (IU/L or IU/mL)	Negative					
**6 months**	1.8 (mU/L or µIU/mL)	<0.1 (µg/L or ng/mL)	≤1 (IU/L or IU/mL)	Negative					
**9 months**	4.1 (mU/L or µIU/mL)	<0.1 (µg/L or ng/mL)	≤1 (IU/L or IU/mL)	Negative					
**12 months**	2.9 (mU/L or µIU/mL)	<0.1 (µg/L or ng/mL)	≤1 (IU/L or IU/mL)	Negative					
**15 months**	1.9 (mU/L or µIU/mL)	<0.1 (µg/L or ng/mL)	≤1 (IU/L or IU/mL)	Negative					
**18 months**	1.5 (mU/L or µIU/mL)	<0.1 (µg/L or ng/mL)	≤1 (IU/L or IU/mL)	Negative					
**2 years**	1.1 (mU/L or µIU/mL)	<0.1 (µg/L or ng/mL)	≤1 (IU/L or IU/mL)	Negative					
**3 years**	1.7 (mU/L or µIU/mL)	<0.1 (µg/L or ng/mL)	≤1 (IU/L or IU/mL)	Negative					
**4 years**	1.4 (mU/L or µIU/mL)	<0.1 (µg/L or ng/mL)	≤1 (IU/L or IU/mL)	Negative					
**5 years**	0.08 (mU/L or µIU/mL)	<0.1 (µg/L or ng/mL)	≤1 (IU/L or IU/mL)	Negative	Hypermetabolic activity in the right upper lung, the skin lesion, and a lymph node in the right anterior lower neck	Metastatic PTC in skin lesion; two benign LNs	Therapeutic dose: 108.7 mCi	Uptake in thyroid bed; no distant avidity	
**5 years, 2 months**	0.15 (mU/L or µIU/mL)	<0.1 (µg/L or ng/mL)	≤1 (IU/L or IU/mL)	Negative					Resolution of right upper lung nodule
**5 years, 6 months**	0.09 (mU/L or µIU/mL)	<0.1 (µg/L or ng/mL)	≤1 (IU/L or IU/mL)	Negative					
**6 years**	0.07 (mU/L or µIU/mL)	<0.1 (µg/L or ng/mL)	≤1 (IU/L or IU/mL)	Negative					

Abbreviations: µg/L, micrograms per liter; µIU/mL, micro–International Units per milliliter; CT, computed tomography; IU/L, International Units per liter; IU/mL, International Units per milliliter; LN, lymph node; mU/L, milliunits per liter; ng/mL, nanograms per milliliter; PET/CT, positron emission tomography/computed tomography; PTC, papillary thyroid carcinoma; RAI-131, radioactive iodine-131; Tg, thyroglobulin; TgAb, thyroglobulin antibodies; TT, total thyroidectomy; US, ultrasound; WBS, whole-body scan.

^*a*^Normal TSH range: 0.5-3.0 mU/L or 0.5-3.0 µIU/mL.

^*b*^Normal Tg range: < 0.1 µg/L or <0.1 ng/mL.

^*c*^Normal TgAb range: ≤ 1 IU/L or ≤1 IU/mL

**Figure 2.**
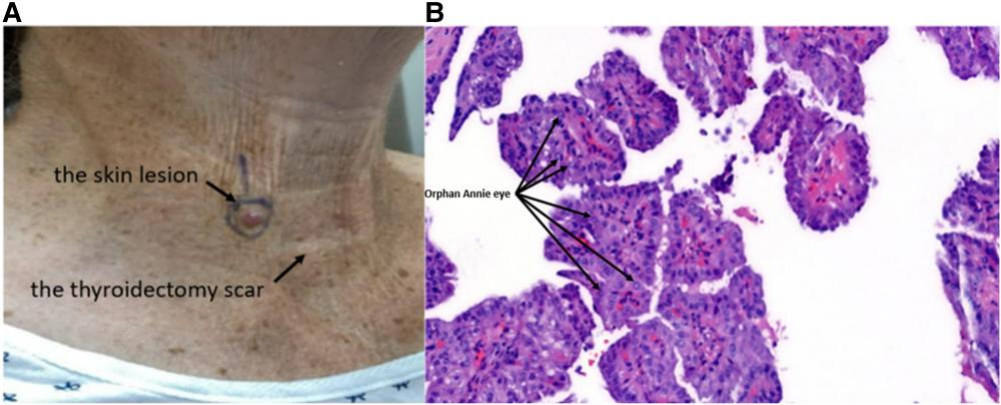
A solitary, well-circumscribed, raised lesion located on the anterior aspect of the lower neck above the total thyroidectomy scar. The lesion is erythematous, dome-shaped, and smooth-surfaced, measuring approximately 0.8 cm in diameter (A). Histopathological examination of the shave biopsy stained with hematoxylin and eosin reveals tumor cells arranged in papillary structures with fibrovascular cores. these cells exhibit characteristic nuclear features, including enlarged nuclei with cleared chromatin (“Orphan Annie eye”), nuclear grooves, and occasional intranuclear inclusions, confirming the diagnosis of metastatic papillary thyroid carcinoma (B).

Five years after diagnosis, the patient presented with a small, painless lesion in the lower right neck (Fig. 2A). Dermatological evaluation and shave biopsy confirmed metastatic PTC (Fig. 2B). TSH was 1.3 mU/L or 1.3 µIU/mL (normal range: 0.5-3.0 mU/L or 0.5-3.0 µIU/mL). Tg level was <0.1 µg/L or <0.1 ng/mL (normal range: < 0.1 µg/L or <0.1 ng/mL). Tg antibody (TgAb) level was ≤1 IU/L or ≤1 IU/mL (normal range: ≤ 1 IU/L or ≤1 IU/mL).

**Figure 3.**
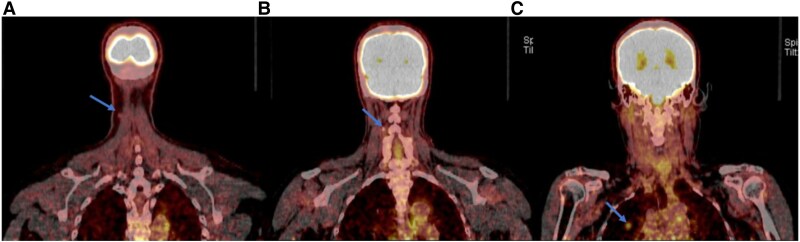
Positron emission tomography/computed tomography imaging from the skull to mid-thigh reveals hypermetabolic activity in the cutaneous lesion in the right anterior lower neck (arrow) (A), the lymph node lesion in the right anterior lower neck (arrow) (B), and the nodule in the right upper lobe of the lung (arrow) (C).

Neck ultrasound showed no evidence of gross recurrence or suspicious lymphadenopathy. Positron emission tomography/computed tomography (PET/CT) imaging identified 2 fluorodeoxyglucose-avid lesions in the right anterior lower neck, 1 corresponding to the cutaneous lesion (Fig. 3A) and the other to an adjacent lymph node (Fig. 3B). Additionally, a hypermetabolic nodule was detected in the right upper lobe of the lung (Fig. 3C). Given the new diagnosis of skin metastasis, the patient's levothyroxine dose was adjusted to achieve TSH suppression < 0.1 mU/L or < 0.1 µIU/mL (normal range: 0.5-3.0 mU/L or 0.5-3.0 µIU/mL).

## Treatment

The patient subsequently underwent excisional removal of the skin lesion and 2 adjacent lymph nodes, which was successfully completed without any complications. Histopathological examination confirmed metastatic PTC in the skin lesion, measuring 0.5 × 0.9 cm. The 2 excited lymph nodes were benign. Following these findings, the American Thyroid Association risk of recurrence was reclassified as high, the response to therapy was reconsidered as structurally incomplete, and RAI ablation was recommended. The patient received 108.7 mCi of RAI-131 therapy following stimulation with recombinant human TSH approximately 2 months later. After skin lesion removal, TSH was measured at 0.08 mU/L or 0.08 µIU/mL (normal range: 0.5-3.0 mU/L or 0.5-3.0 µIU/mL). Tg and TgAb remained undetectable.

Posttherapy whole-body scan revealed uptake in the thyroid bed, consistent with remnant tissue, but no additional foci of uptake to suggest persistent or recurrent metastasis, including in the previously identified lung nodule. Given the lack of iodine avidity in the right upper lung lesion detected on the previous PET/CT imaging, the patient was referred to a lung nodule clinic for further evaluation.

**Figure 4.**
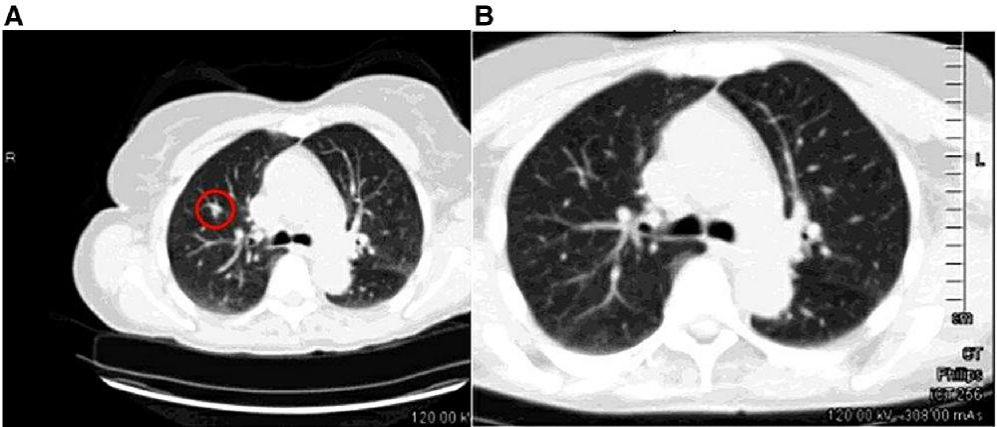
The nodule in the right upper lobe, previously detected on the CT component of the PET/CT scan (circled) (a), is no longer visible on the follow-up CT scan (B). Abbreviations: CT, computed tomography; PET, positron emission tomography.

A CT-guided biopsy was initially recommended; however, subsequent noncontrast chest CT imaging obtained 2 months later demonstrated complete resolution of the lung nodule, suggesting a benign etiology or transient inflammatory process and eliminating the need for further intervention (Fig. 4A and 4B).

## Outcome and Follow-up

At a 2-year follow-up, the patient remained clinically stable with no new cutaneous lesions identified during dermatological surveillance. The previously detected lung nodule was not seen on repeated CT imaging. The patient continues long-term surveillance with periodic imaging and laboratory assessments, which have consistently shown no evidence of recurrent disease (Table 1). Levothyroxine suppression therapy has been maintained to keep TSH levels low. At the most recent follow-up, 2 years posttreatment, the patient remained asymptomatic, with undetectable Tg and TgAb levels, and no adverse or unanticipated events were observed.

## Discussion

Cutaneous metastasis from PTC is an exceptionally rare clinical entity and is not commonly reported in the medical literature (Table 2), occurring in fewer than 1% of PTC cases [2]. While the lungs and bones are the most common sites for distant metastases, skin involvement typically reflects widespread disease and portends a poor prognosis [2]. In our case, the development of an isolated cutaneous lesion on the lower right neck 5 years after the initial treatment highlights the complexity of PTC's metastatic behavior and the diagnostic challenges it poses. The presentation of cutaneous metastases from PTC is often subtle and can mimic benign dermatological conditions, leading to diagnostic delays. Typical manifestations include erythematous or violaceous nodules, plaques, or, less commonly, ulcerative lesions [5]. These lesions frequently localize to the head, neck, and scalp regions due to the rich vascular supply, which facilitates hematogenous dissemination [24]. Histopathological evaluation remains the gold standard for diagnosis, with immunohistochemical staining for TTF-1 and Tg providing definitive evidence of thyroid origin [24]. In our case, a shave biopsy confirmed the diagnosis of metastatic PTC, consistent with previously reported cases. Positivity for TTF-1 and Tg, along with classical histological features such as Orphan Annie eye nuclei, fibrovascular cores, and psammoma bodies, substantiated the thyroidal origin [25, 26]. Advanced imaging techniques, including PET/CT, further delineated the extent of the disease, revealing a hypermetabolic lung nodule that subsequently resolved, highlighting the utility of multimodal imaging in ambiguous cases [25]. The mechanisms underlying skin metastasis in PTC remain incompletely understood. Hematogenous spread is the most accepted pathway, wherein circulating tumor cells are captured by the dermal capillary network and establish metastatic foci [10, 23]. Other potential mechanisms include lymphatic dissemination, direct extension, and iatrogenic implantation, particularly in cases of prior fine-needle aspiration biopsy or surgical procedures [23]. Although needle tract implantation has been reported in rare cases, its role in this patient's metastasis is less likely due to the lesion's location, and there is no direct evidence linking the procedure to the tumor's spread [25]. Cutaneous metastasis from PTC is often associated with advanced disease and a limited life expectancy, with a median survival of 8 to 19 months after diagnosis [5, 10]. The prognosis depends on factors such as tumor burden, iodine avidity, and the presence of actionable genetic mutations. In our case, the solitary nature of the metastasis and the absence of additional lesions on posttherapy imaging suggest a more favorable prognosis than typically observed [10, 23]. This case report and literature review discuss survival outcomes and prognosis.

**Table 2.** Case reports of skin metastasis from thyroid cancer

Case	Year	Age/sex	Tumor type	Initial treatment	Skin lesion (location, time since initial surgery)	Spread	Recurrence treatment	Outcome
**Horiguchi [6]**	1984	62/M	c-PTC	Hemithyroidectomy	Scalp (left temporal, 3 years)	bone	Surgery, RAI, radiation	No progression
**Horiguchi** **[6]**	1984	70/F	c-PTC	TT	Head, abdomen, legs (1 year)	None	Surgery	Slow progression
**Doutre [7]**	1988	59/F	c-PTC	TT and RAI	Scalp (3 nodules, 8 years)	bone, lungs	Not reported	Died
**Elgart [8]**	1991	59/M	c-PTC	Subtotal TT and RAI	Scalp (parietal, 3 years)	Femur, chest, lungs	Surgery	Not reported
**Ronga [9]**	2006	59/F	c-PTC	TT	Neck (scar, 20 years)	None	Surgery	No recurrence
**Avram [10]**	2007	63/M	Metastatic PTC	TT, RAI, EBRT	Face, scalp (17 years)	Lungs, LNs, bones, choroid	Surgery, RAI	Progressed
**Bucerius [11]**	2008	57/F	c-PTC	Hemithyroidectomy, Completion TT, RAI	Thigh, thorax	Choroid, lung, LNs	Surgery	Died
**De Giorgi [12]**	2009	86/M	c-PTC	TT and RAI	Supraclavicular (left, 12 years)	Lungs	Surgery	No recurrence
**Shon [13]**	2010	68/M	Hurthle cell	Unknown	Scrotum, chin	Unknown	Unknown	Unknown
**Camacho [14]**	2010	47/M	FTC	TT and RAI	Nose	Bone, cervical, lung	Surgery (nasal nodule)	Unknown
**Kwon [15]**	2014	55/F	c-PTC	TT and RAI	Neck (movable, 3 years)	Unknown	Surgery	Recurrence resected
**Reusser [4]**	2014	95/M	Unknown	Partial TT	Neck (bleeding ulcer, 9 years)	Neck LN	Unknown	Unknown
**Jehangir [16]**	2015	65/F	FTC	TT and RAI	Temporal, parietal	Skull masses	Surgery	No recurrence
**Farina [3]**	2016	78/F	c-PTC	TT and RAI	Scalp (right parietal, 6 years)	Pancreas, bone	Surgery, RAI, sorafenib	Stable
**Soylu [17]**	2017	83/F	c-PTC	TT and RAI	Neck (movable, 3 years)	None	Surgery	No recurrence
**Sindoni [18]**	2018	47/M	c-PTC	TT and RAI	Neck (pimple-like, 11 years)	Unknown	Surgery, RAI	No recurrence
**Lira [19]**	2019	55/F	Follicular adenoma	TT	Neck (papule, 6 years)	Unknown	Surgery	No recurrence
**Cheng [20]**	2020	65/F	c-PTC	TT and LN dissection	Supraclavicular	Lungs, cervical LNs	Surgery, EBRT	Died
**Liu [21]**	2022	57/M	c-PTC	TT and LN dissection	Shoulder	Cervical, supraclavicular, axillary LNs	Surgery	Unknown
**Alwhaid [1]**	2022	70/F	fv-PTC	TT	Scalp, arm (30 years)	Lungs, bones	Sorafenib, palliative care	Died
**Tanal [22]**	2022	63/F	c-PTC (undetected initially)	Subtotal + completion TT	Neck (14 years)	Bilateral lungs	Surgery, RAI	Progressed
**Choi [23]**	2023	44/F	c-PTC	TT and RAI	TT Scar (10 years)	Unknown	Surgery, RAI	No recurrence
**Chu [24]**	2024	23/F	c-PTC (BRAF V600E)	Hemithyroidectomy, LN Dissection	Neck (2 lesions, 2.5 years)	unknown	Surgery	No recurrence

Abbreviations: BRAF, B-rapidly accelerated fibrosarcoma; c-PTC, classic papillary thyroid carcinoma; EBRT, external beam radiation therapy; F, female; FTC, follicular thyroid carcinoma; fv-PTC, follicular variant papillary thyroid carcinoma; LN, lymph node; M, male; RAI, radioactive iodine ablation; TT, total thyroidectomy.

Management of cutaneous metastases in PTC includes surgical excision, RAI therapy, and systemic treatments such as tyrosine kinase inhibitors. In iodine-avid cases, RAI remains the cornerstone of systemic therapy, as it can target residual and metastatic disease effectively [5, 24, 27]. However, the presence of B-rapidly accelerated fibrosarcoma (*BRAF*) mutations in PTC has been associated with reduced iodine avidity and a more aggressive clinical course, necessitating additional therapeutic approaches such as tyrosine kinase inhibitors or combination therapies targeting the mitogen-activated protein kinase and phosphoinositide 3-kinase/protein kinase B pathways [28, 29]. Our patient underwent surgical removal of the skin lesion and 2 benign lymph nodes and RAI therapy. Posttherapy whole-body scans revealed uptake confined to the thyroid bed, suggesting effective management of the metastatic lesion [23].

The role of genetic mutations such as *BRAFV600E* and telomerase reverse transcriptase promoter mutations in PTC has gained significant attention. These mutations are linked to aggressive disease phenotypes, reduced iodine avidity, and distant metastases, including rare sites such as the skin [6, 27]. While genetic testing was not performed in this case, the integration of molecular profiling into routine practice could improve risk stratification and guide personalized therapies. For instance, dual inhibition of the mitogen-activated protein kinase and phosphoinositide 3-kinase/protein kinase B pathways has shown promise in preclinical models, inducing apoptosis and restoring iodine uptake in refractory cases [28].

In conclusion, this case underscores the importance of long-term surveillance in patients with PTC, even among those initially classified as low risk. The rare occurrence of isolated cutaneous metastasis highlights the complexity of PTC's metastatic behavior and the need for a multidisciplinary approach to achieve optimal management. Future research should focus on elucidating the molecular mechanisms driving atypical metastatic patterns and exploring novel therapeutic strategies to improve outcomes for this subset of patients.

## Learning Points

- Isolated skin metastases from PTC are rare and often signify advanced disease.
- Histopathological evaluation and immunohistochemical staining are critical for diagnosis.
- Multimodal imaging aids in delineating disease extent and planning treatment.
- Long-term surveillance is vital, even in low-risk PTC cases.
- Molecular profiling may inform prognosis and guide personalized therapy.

## Data Availability

Original data generated and analyzed for this case report are included in this published article.
